# The Effect of Corneal Refractive Surgery on Glaucoma

**DOI:** 10.1155/2017/8914623

**Published:** 2017-04-09

**Authors:** Vassilios Kozobolis, Aristeidis Konstantinidis, Haris Sideroudi, G. Labiris

**Affiliations:** University Eye Clinic, University Hospital of Alexandroupolis, 68131 Alexandroupolis, Greece

## Abstract

Laser-assisted refractive procedures have become very popular in the last two decades. As a result, a “generation” of patients with altered corneal properties is emerging. These patients will require both cataract extraction and glaucoma follow-up in the future. Since the glaucoma examination largely depends on the corneal properties, the reshaped postrefractive surgery cornea poses a challenge in the diagnosis, follow-up, and management of the glaucomatous patient. In order to overcome this problem, every patient who is planned to undergo corneal refractive surgery must have a thorough glaucoma examination in order for the ophthalmologist to be able to monitor their patients for possible glaucoma development and/or progression. Some examinations such as tonometry are largely affected by the corneal properties, while others such as the evaluation of the structures of the posterior pole remain unaffected. However, the new imaging modalities of the anterior segment in combination with the most recent advances in tonometry can accurately assess the risk for glaucoma and the need for treatment.

## 1. Introduction

Laser-assisted refractive corrections constitute a large part of the ophthalmic surgeries that take place every year. It is estimated that about 4 million refractive procedures were performed in 2014 throughout the world. On the other hand, glaucoma is an optic neuropathy, the incidence of which is increasing steadily over time. In 2013, the number of glaucoma patients was estimated at about 64.3 million and is expected to reach 118.3 million by 2040 [1]. Given the frequency of refractive corrections and the incidence of glaucoma in the general population, it becomes necessary for the ophthalmologist to assess the risks of a laser-assisted refractive operation in a glaucoma patient or a patient at a high risk of developing glaucoma in the future.

## 2. Preoperative Assessment

Every patient who is planned to undergo laser-assisted refractive correction should be evaluated for the risk of developing glaucoma in the future. Among others, the following factors should be taken into consideration.

### 2.1. Family History of Glaucoma

Epidemiological studies have shown that people with familiar predisposition for glaucoma (especially with first-degree relative) have increased risk of developing ocular hypertension (OHT) and glaucoma. Moreover, these individuals tend to develop glaucoma/OHT at a younger age than the general population [2, 3]. The assessment of the presence of glaucoma in a patient's family is therefore of great importance in order to estimate the risk of developing glaucoma in the future.

### 2.2. Intraocular Pressure (IOP)

Elevated IOP remains the most important, modifiable, risk factor for developing glaucoma [4, 5]. However, a single IOP measurement is not sufficient to assess the actual risk of glaucoma, especially when there are other coexisting risk factors. A better understanding of the characteristics of the IOP (average IOP, highest reading, and diurnal fluctuation) is achieved by taking more than one measurements of the IOP in a 24-hour period. Large diurnal fluctuations of the IOP and/or IOP asymmetry between the two eyes are an indication of increased likelihood of developing glaucoma [6, 7].

### 2.3. Myopia

Myopia is a risk factor of developing glaucoma, and most patients undergoing refractive surgery are potentially glaucoma patients. High myopes (>6.00 D) have a higher risk [8]. Furthermore, tilted discs and peripapillary atrophy are more often seen in high myopes and this can complicate the clinical assessment of the glaucomatous optic neuropathy and monitor changes of the disc structure and the retina over time. As the modern imaging tools do not include high myopes in their database (high myopes are rather excluded), the measurements that they provide are unreliable. In these cases, preoperative photography of the disc is of great value.

### 2.4. High Vertical Cup-to-Disc Ratio

Although the cup-to-disc ratio in the vertical axis shows great diversity, a high vertical C/D ratio is a risk factor of developing glaucoma [9]. The parameters of the optic disc and the thickness of peripapillary layer of nerve fibers play a pivotal role in the postoperative follow-up of patients who have undergone refractive surgery.

### 2.5. Central Corneal Thickness

It is well known that a thin cornea is not only a limiting factor for laser-assisted surface ablations but also an independent risk factor for developing glaucoma [9, 10].

### 2.6. Race

People of Afro-Caribbean origin develop open-angle glaucoma more often and at an earlier age than white people [11], although this may be partly due to the fact that black people have thinner corneas [12].

### 2.7. Other Ophthalmic Diseases

Pseudoexfoliation syndrome [13–15] and pigment dispersion syndrome [16] are known risk factors for secondary open-angle glaucoma. A study of 12 patients (22 eyes) with pigment dispersion syndrome showed that its presence does not affect the results of refractive surgery, but the authors indicate that the final refractive outcome in patients who receive topical antiglaucoma medication before surgery is less predictable and the healing process of the corneal wound can last longer [17].

### 2.8. Hypermetropia

Hypermetropes are more likely to have narrow anterior chamber angles and a case of acute angle closure after LASIK in a hypermetropic patient has been reported [18]. Preoperative gonioscopy will help the surgeon to recognize patients with narrow angles.

### 2.9. Previous Antiglaucoma Procedure

Photorefractive keratectomy (PRK) is the safest surgical option in patients with previous antiglaucoma filtering operation [19]. The creation of the corneal flap with the mechanical keratome or the femto-second laser (docking) during LASIK may damage the filtering bleb and compromise its function. The new refractive lenticule extraction surgery still requires docking of the femto-laser operating system on the eye and should be carefully used in eyes with thin blebs.

### 2.10. Visual Fields

Preoperative visual fields help the surgeon identify the following:

- The presence of established glaucomatous damage
- The extent of glaucomatous damage
- The risk of developing glaucoma. Patients with high PSD have a greater chance of developing glaucoma, even in the absence of visual fields scotomas [20]. Consequently, the preoperative examination of the visual fields, especially in patients with predisposing factors for glaucoma, is a useful tool for the future monitoring of refractive patients.

### 2.11. Modern Imaging Modalities

Modern imaging methods (OCT, HRT, and GDx) provide quantitative analysis of the peripapillary optic nerve fibers at a particular distance from the center of the optic disc. They also provide information for several structural parameters of the optic nerve head. In order to differentiate between the disc cup and the nerve fiber rim, they use a reference plane. The structures above the reference plane are read as the rim of the nerve fibers, and the structures below it are recognized by the device as the disc cup. The advantages include objective and reproducible measurements that can be compared with future measurements. The disadvantage is that their databases (although constantly enriched) include limited number of people, while “unusual” discs (tilted, high ametropias) are excluded from the databases. Unfortunately, many candidates for refractive surgery have optic discs with “unusual” appearance that cannot be meaningfully compared with the “normal” optic discs of the databases. In these cases, the digital photographing of the optic disc and the comparison with future photos will give valuable information about the changes of both the optic nerve and retinal nerve fibers.

The red-free imaging of the optic disc is as valuable in differentiating between normal and glaucomatous patients as the OCT (optical coherence tomography), the SLP (scanning laser polarimetry), and the CSLO (confocal scanning laser ophthalmoscope) [21–24].

## 3. Intraoperative Risk Factors for Glaucoma Progression

During the corneal flap creation in LASIK, the intraocular pressure can go as high as 90 mmHg [25, 26]. The effect of high IOP on the vascular perfusion of the retina has been studied experimentally in pigs but not in glaucoma patients. Research has shown that increased IOP significantly lowers the blood flow through the vessels. The point at which the flow stops completely depends not only on the level of the IOP but also on the blood pressure as well [27]. LASIK surgery does not seem to affect the structure and function of the optic nerve (visual fields, color perception, contrast sensitivity, and pupillary reflex) despite the transient significant elevation of the IOP during surgery [28]. Additionally, it has not been shown that the LASIK affects the structure of the optic nerve or the thickness of the layer of nerve fibers [29–31]. Some studies have reported a reduction of the nerve fiber layer after LASIK [32] with the SLP technology used by the GDx machines, but these effects are probably due to the change of the corneal birefringence and are not real damage of the retinal nerve fibers [33–35]. The new GDx machines with enhanced corneal compensator (ECC) seem to overcome this issue [36].

However, cases of ischaemic optic neuropathy following LASIK and epi-LASIK that can cause permanent damage to the optic nerve have been reported [37–39].

The visual fields, as assessed by automated static perimetry, do not seem to be affected after refractive surgery in the glaucoma and normal population [40]. Nevertheless, there have been reports of visual field deterioration in people with and without glaucoma [41, 42]. It is possible that a small group of glaucoma patients are prone to develop optic nerve damage following an elevation of the IOP during LASIK, but the visual field defects are either very mild or masked by the learning effect of the visual field examination [40]. There have also been reports of loss of the contrast sensitivity and scotoma development from the transition zone [43, 44].

In summary, although the sudden increase of the IOP during LASIK surgery does not appear to affect significantly the structure and function of the optic nerve, it is recommended that the PRK is the preferred method of refractive surgery in the case of the glaucoma patient [19].

## 4. Postoperative Patient Assessment

### 4.1. The Effect of the Central Corneal Thickness on the Measurement of the IOP

Goldmann applanation tonometry is still the gold standard method of measuring the IOP. This tonometer was first described by Hans Goldmann and Theo Schmidt in 1957 [45], and it is based on the Imbert-Fick principle.

Both PRK [46–48] and LASIK [49–52] cause a reduction of the postoperative IOP. This reduction (and consequently the clinical underestimation of the actual postoperative IOP) depends on the depth of the ablation and the preoperative IOP. The deeper the ablation and the higher the preoperative IOP, the greater the postoperative reduction of the IOP will be. In addition, the myopic refractive surgery causes larger underestimation of IOP compared to the hypermetropic corrections which are thought to cause negligible IOP change. The postoperative reduction of the IOP is due to the thinning of the corneal stroma, the change in corneal curvature, the instability of the corneal flap (LASIK) [50, 51], and the removal of the Bowman's layer (PRK) [46]. In order to calculate the reduction of the postoperative IOP, Kohlhaas et al. [51] proposed an algorithm that computes the actual IOP after myopic LASIK [IOP (real) = IOP (measured) + (540 − CCT)/71 + (43 − *K* − value)/1.7 + 0.75 mm Hg], where IOP (real) is the actual IOP; IOP (measured) is the measured IOP; CCT is the central corneal thickness postoperatively; and *K* is the average of keratometry readings postoperatively. However, there is not still a commonly accepted algorithm that can calculate with high accuracy the level of the actual postoperative IOP [52].

In order to overcome the problem of the postoperative IOP underestimation with the Goldmann tonometer, some authors suggest that the measurement (in myopic eyes) is done in the periphery of the cornea where less corneal tissue is removed.

The pneumatonometer applanates a smaller area of the corneal surface than the Goldmann tonometer does. It also records a lower IOP postoperatively [50, 53, 54], but some writers argue that the underestimation is lower than that of the Goldmann tonometer [48, 55].

Tonopen is a popular applanation tonometer based on the Mackay-Marg principle. Compared to the Goldmann tonometer, its measurements are less influenced by the thinning of the stroma and the reduction of the corneal curvature [56]. The advantage is that it can record IOP measurements from the periphery of the cornea where the measurement is considered more representative of the true intraocular pressure as the stromal thinning and the change of curvature are smaller there [57–59].

The Pascal Dynamic Contour Tonometer (DCT) is based on contour matching. Its advantage lies on the fact that the measurements are not influenced by the viscoelastic properties of the cornea. It is generally thought that the DCT understates to a lesser extent of the IOP compared to the Goldmann tonometer after both LASIK and PRK [60, 61]. It is also more accurate than the pneumatonometer [62, 63].

### 4.2. Effect on the Corneal Viscoelastic Properties

Several studies have shown that the viscoelastic properties of the cornea are reduced after LASIK *και* PRK [64–66] because of the corneal thinning and the creation of the corneal flap. IOPcc is affected to a lesser extent than the IOPg [65], while the IOPg and the IOP estimations with the Goldmann tonometer are reduced to the same extent [67].

The corneal viscoelastic properties have shown a reduction after LASIK [68], which can be attributed to the corneal thinning and the formation of the corneal flap. The IOP measurement with the Corvis ST seems to underestimate the IOP reduction less than the IOPg reading of the ORA and the IOP measurement with the Goldmann tonometer. The postoperative estimation of the IOP with the ORA's IOPcc reading and Corvis ST are the most accurate methods.

The biomechanical properties of the cornea can also be measured with the Corvis ST tonometer which applanates the cornea with a jet of air and the surface deformation is recorded by a high speed and high resolution Scheimpflug camera [69]. The deformation pattern as captured by the Scheimpflung also changes after corneal refractive surgery which is attributed to the corneal changes incurred by the stromal ablation and flap formation [70].

### 4.3. Interface Fluid Syndrome, IFS

This syndrome is due to fluid accumulation between the corneal flap and the underlying stroma after LASIK surgery. This fluid may act as a “cushion” resulting in a falsely low IOP reading as measured with the Goldmann tonometer, while the IOP with other tonometers may be measured correctly high [71–75]. If this condition remains undiagnosed and the IOP is not assessed correctly with a different type of tonometer (other than the Goldmann tonometer), visual capacity may be threatened due to a continuous deterioration of the glaucomatous damage. Pham et al. [76] report a case of this syndrome that appeared 6 years after LASIK following an eye injury with a substantial increase of the IOP. The patient showed signs of ischemic optic neuropathy as the rise of the IOP were not detected by the Goldmann tonometer. Rehany et al. describe a patient with high IOP after LASIK where the Tonopen and the Goldmann tonometers failed to unveil a high IOP which was measured correctly with the Schiøtz tonometer [77]. Najman-Vainer et al. [78] and Shaikh et al. [79] warn even for end-stage glaucoma risk if the IOP is not measured correctly and the ophthalmologist does not rely on functional tests (visual field). This syndrome should be distinguished from the diffuse lamellar keratitis (DLK) as it does not respond to topical steroids and requires treatment aqueous suppressants.

### 4.4. Steroid Responders

The international literature [80] has shown that the use of topical steroids postoperatively can lead to a significant rise of the IOP especially in patients with the following:

- Primary open-angle glaucoma (POAG)
- Glaucoma suspects
- People with first-degree relatives suffering from POAG
- Diabetes mellitus type Ι
- High myopia
- People with a previous episode of steroid responsiveness
- Patients with rheumatic diseases (e.g., rheumatoid arthritis)
- Advanced age.

Increased IOP leads to a spectrum of clinical manifestations in the cornea that ranges from a simple rise of the IOP to pressure induced stromal keratitis (PISK) and to IFS [81, 82]. In the early stages of the IOP rise, there is stromal swelling which causes corneal haze. The corneal swelling then leads to fluid accumulation between the corneal flap and the stroma [83, 84]. If there is fluid accumulation under the flap, the IOP should be measured with a tonometer other than the Goldmann tonometer so as not to miss the diagnosis of IFS. In this case, topical steroids must be stopped and treatment with topical aqueous suppressants must be initiated.

### 4.5. Corneal Permeability after Refractive Surgery

Studies in patients have shown that the corneal permeability increases after PRK and LASIK surgery. Specifically, the corneal permeability to fluorescein increased the first 2 months postoperatively and then decreased gradually from the second until the sixth month postoperatively when it returned to normal levels. Indeed, the deeper the ablation, the higher the corneal permeability [85]. Chung and Feder [86] also noted that three months after LASIK instillation of tropicamide drops caused greater pupil mydriasis 10, 15, and 20 minutes after instillation. Unlike the above reports, the experimental PRK and LASIK in hares caused nonsignificant increase of corneal permeability to timolol 1 month after surgery [87]. The concentration of timolol in the aqueous was measured by liquid chromatography.

### 4.6. Topical Antiglaucoma Medication after Refractive Surgery

Unfortunately, little evidence exists about the effectiveness of the topical antiglaucoma drops in refractive patients. The combination of timolol 0.5% twice a day and dorzolamide 3 times a day is more effective in lowering the IOP after PRK in ocular hypertensive patients compared to timolol twice daily alone or dorzolamide 3 times a day alone [88]. Latanoprost and timolol have the same hypotensive effect in ocular hypertension due to steroid responsiveness [89].

## 5. Conclusions

The preoperative assessment of glaucoma patients who are candidates for refractive surgery should be based on a set of tests which starts from the family history, IOP measuring (even performing a 24-hour IOP phasing in some cases), visual field test, and imaging of the optic nerve and the peripapillary nerve fiber layer. Because age is a strong risk factor [90], it is easily understood that all young refractive patients are potentially glaucoma patients over time. Therefore, the preoperative glaucoma risk assessment should be performed in every patient.

The preoperative imaging of the structures of the posterior pole can be done with digital photography or/and with one of the newer imaging methods (OCT, HRT, and GDx). Fundus photography does not give objective measurements of the structures but enables us to monitor the changes over time even in “unusual” discs (tilted and myopic discs). The other imaging modalities provide detailed measurements of various parameters of the structures of the posterior pole. They also compare the parameters of each individual patient to a database of normal individuals. However, these comparisons may not be entirely reliable in patients with “unusual” discs as occurs in many myopic patients who are excluded from the database of these machines.

The correct measurement of the postoperative IOP is an important challenge for the ophthalmologist. The changes in the corneal thickness, curvature, viscoelastic properties, and the creation of the corneal flap (in LASIK and epi-LASIK) make the assessment of IOP with the Goldmann tonometer unreliable. The clinician should not rely only on the IOP measurement for the diagnosis and monitoring of glaucoma suspects or true glaucoma patients. Visual field tests and imaging of the optic nerve are needed to monitor these patients. In order to accurately estimate the true IOP, the measurements should be done with the Tonopen (which has a smaller applanation surface than the Goldmann tonometer) from the periphery of the cornea or with the DCT whose measurements are not affected by the viscoelastic properties of the cornea. The ORA's IOPcc, which is less affected by the corneal changes, and the Corvis ST are thought to estimate more accurately the true level of the IOP following a refractive procedure.

The clinician should always bear in mind the possible diagnosis of PISK which has a similar clinical picture with DLK but does not respond to topical steroids and should be treated with aqueous suppressants. PISK can be complicated by fluid accumulation under the corneal flap, in which case, the Goldmann tonometer can significantly underestimate the true IOP. As a consequence, the IOP must be monitored with more than one tonometer.

In summary, every young glaucoma patient should be treated as a future glaucoma patient and baseline tests should be carried out preoperatively. In this way, the ophthalmologist will be able to recognize the development of glaucomatous optic neuropathy in the future.
